# *N*-acyl Homoserine Lactone-Producing *Pseudomonas putida* Strain T2-2 from Human Tongue Surface

**DOI:** 10.3390/s131013192

**Published:** 2013-09-30

**Authors:** Jian-Woon Chen, Shenyang Chin, Kok Keng Tee, Wai-Fong Yin, Yeun Mun Choo, Kok-Gan Chan

**Affiliations:** 1 Division of Genetics and Molecular Biology, Institute of Biological Sciences, Faculty of Science, University of Malaya, Kuala Lumpur 50603, Malaysia; E-Mails: cjw246@hotmail.com (J.-W.C.); shenyang86@yahoo.com (S.C.); yinwaifong@yahoo.com (W.-F.Y.); 2 Centre of Excellence for Research in AIDS (CERiA), Department of Medicine, Faculty of Medicine, University of Malaya, Kuala Lumpur 50603, Malaysia; E-Mail: k2tee@um.edu.my; 3 Department of Chemistry, Faculty of Science, University of Malaya, Kuala Lumpur 50603, Malaysia; E-Mail: ymchoo@um.edu.my

**Keywords:** autoinducer, bioreporter, *luxS*, *N*-acylhomoserine lactone, oral buccal cavity, quorum quenching, quorum sensing

## Abstract

Bacterial cell-to-cell communication (quorum sensing) refers to the regulation of bacterial gene expression in response to changes in microbial population density. Quorum sensing bacteria produce, release and respond to chemical signal molecules called autoinducers. Bacteria use two types of autoinducers, namely autoinducer-1 (AI-1) and autoinducer-2 (AI-2) where the former are *N*-acylhomoserine lactones and the latter is a product of the *luxS* gene. Most of the reported literatures show that the majority of oral bacteria use AI-2 for quorum sensing but rarely the AI-1 system. Here we report the isolation of *Pseudomonas putida* strain T2-2 from the oral cavity. Using high resolution mass spectrometry, it is shown that this isolate produced *N*-octanoylhomoserine lactone (C8-HSL) and *N*-dodecanoylhomoserine lactone (C12-HSL) molecules. This is the first report of the finding of quorum sensing of *P. putida* strain T2-2 isolated from the human tongue surface and their quorum sensing molecules were identified.

## 1. Introduction

Proteobacteria communication is commonly known as quorum sensing (QS) which regulates diverse bacterial phenotypes including those regulating virulence determinants [1–6]. QS usually involve production and sensing of the signaling molecules which will then bind to a cognate receptor [7]. The signal molecule-receptor complex will then be able to moderate a myriad genes regulation. Most Gram-negative bacteria produce a type of autoinducer molecule called *N*-acyl homoserine lactones (AHLs) which are produced by AHL synthase. Autoinducer-2 (AI-2) is a signaling molecule which is a product of the *luxS* gene [8–10] and AI-2 represents the only QS mechanism found in both Gram-negative and Gram-positive bacteria [11–13]. Both AHL and AI-2 are important signal molecules among different species in a polymicrobial community [14–23].

The oral cavity is a very unique environment where the oral microbiome consists of several hundreds of bacteria genera [24]. It has been reported that bacteria can be found in new orthodontic buccal tubes [25]. In spite of the microbial abundance in the oral cavity, it is noteworthy that nearly all these oral bacteria exhibit AI-2 QS instead of the AHL-based system. In our recent work, we isolated two AHL-producing oral bacteria namely *Enterobacter* sp. and *Klebsiella pneumoniae* [26,27]. It has been reported that AI-2 is vital for the biofilm formation in *K. pneumoniae* [28]. Similarly, AI-2 also regulates expression of virulence factors [29–31].

AHLs are QS signalling molecules in Proteobacteria, and are produced by an AHL synthase (LuxI) so that when signaling molecules bind to LuxR protein, this AHL-luxR complex will be used to regulate QS-based gene expression [31–34]. When the concentration of these AHLs reaches the threshold level, the AHL-luxR complex will regulate a set of genes which occur in a population density-dependent manner, leading to population driven changes in several functions including virulence determinants, antibiotic production, bioluminescence, and biofilm formation [35]. QS bacteria have been isolated from various sources and habitats, including the human body [36–45].

## 2. Experimental Section

### 2.1. Bacterial Strains

In this work, *Escherichia coli* [pSB401] [46] and *Agrobacterium tumefaciens* NTL4 (pZLR4) [47] were used as short and long chain AHLs biosensors, respectively. While the former produces the bioluminescence in the presence of exogenously supplied short chain AHLs, the latter turns blue on AB agar supplemented with X-gal (60 μg/mL, final concentration) and medium and long chain AHLs. Routinely, *A. tumefaciens* NTL4 (pZLR4) was cultured in AB medium or AB agar (solidified with bacto-agar at 1.5 g/l00 mL), supplemented with gentamicin (150 μg/mL) and glucose (0.5% w/v) according to previously reported work [47]. To detect AHL molecules with *A. tumefaciens* NTL4 (pZLR4), AB agar without gentamicin was supplemented with X-gal. All other bacteria were routinely cultured in Luria–Bertani (LB) medium (in grams per 100 mL: tryptone, 1; NaCl, 0.5; yeast extract, 0.5), broth or agar (solidified with bacto-agar at 1.5 g/l00 mL), buffered to pH 5.5 with 50 mM 3-(*N-*morpholino)propanesulfonic acid (MOPS) to prevent lactonolysis due to basic condition [48]. Where necessary, growth media were supplemented with ampicillin (100 μg/mL). *A. tumefaciens* NTL4 (pZLR4) was grown at 28 °C, whereas *E. coli* DH5α, *E. coli* [pSB401] and oral bacteria were grown at 37 °C.

### 2.2. Enrichment of Bacteria from Tongue Surface Debris

This study was approved by the Ethics Committee of the Faculty of Dentistry (University of Malaya). A tongue surface debris sample was collected from healthy individuals in 2008 at the Faculty of Dentistry. Samples from the posterior dorsum surface of the tongue were taken by gentle scraping the tongue surface using a sterile stainless steel tongue scraper. The materials on the scraper were quickly placed into sterile saline (300 μL) containing in a sterile tube. Oral bacteria used in this study were isolated as previously described [26,27] using KG medium [49]. To obtained single pure colony, bacterial culture was spread on LB agar by repeated streaking. Bacterial colony labelled as T2-2 was selected for further studies.

### 2.3. Strain Identification

Bacteria DNA extraction, purification, manipulations and amplification 16S rDNA gene by polymerase chain reaction (PCR) were carried out as reported [50]. We used universal primer pairs 27F and 1525R to amplify the 16S rDNA genes from the purified bacterial genomic DNA [51]. Universal primers T7, SP6, and internal primers previously designed to anneal to internal target regions of the 16S rDNA were used as reported previously [52]. Nucleotide sequences were aligned and phylogenetic analysis with 1,000 re-samplings was performed as reported previously [26,27] to ensure robustness and topology of the tree constructed.

### 2.4. Extraction of AHLs from Bacterial Culture Supernatants

Overnight grown bacterial culture (1 mL) was inoculated into LB broth (100 mL) and cells were grown to an OD_600_ of 1.0. The spent supernatant was extracted vigorously twice with ethyl acetate (100 mL). After settling into two layers, the organic phase was collected in a separation funnel, dried over excess anhydrous magnesium sulphate, filtered through filter paper, and the extract was evaporated to completely dryness. To dissolve the extracted AHLs, 100 μL of acetonitrile was added to the extracts, mixed well and kept at −20 °C.

### 2.5. Measurement of Bioluminescence

To measure bioluminescence, we followed the method reported previously [26] where *E. coli* [pSB401] was grown overnight and diluted with sterile LB broth to OD_600_ 0.01, and these cells (200 μL) were added to each well of a 96-well optical bottom microtitre plate. AHL extracts from oral bacterium T2-2, AHL solvent (ethyl acetate) (control), PBS buffer (control) were added to the wells containing *E. coli* [pSB401] cells, and incubated at 37 °C for 24 h and measured in luminometer-spectrophotometer. Bioluminescence was measured using a combined automated Tecan luminometer-spectrophotometer (Infinite, Tecan, Männerdorf, Switzerland) essentially as reported before [26]. Growth measurements and bioluminescence were the averages of triplicate experiments. Data were presented as graph Relative Light Units (RLU)/OD_600 nm_ against time, indicating approximate light output per cell.

### 2.6. Mass Spectrometry (MS) Analysis of AHL

High resolution mass spectrometry was performed as reported [27] using an Agilent RRLC 1200 system coupled with an Agilent ZORBAX Rapid Resolution HT column (100 mm × 2.1 mm, 1.8 μm particle size). Mass spectrometry was done using an Agilent 6500 Q-TOF system and mass spectrometry ESI-MS and ESI-MS/MS analysis conditions (60 °C, flow rate 0.3 mL/min, with injection volume 20 μL. Mobile phases A and B were 0.1% v/v formic acid in water and 0.1% v/v formic acid in acetonitrile, respectively and the mobile phases gradient profile; ESI-positive mode, probe capillary voltage set at 3,000 V; desolvation temperature 350 °C; sheath gas 11 mL/h; and nebulizer pressure 50 psi) were performed essentially as previously described [27]. We used nitrogen gas as the collision gas in the collisionally induced dissociation mode for the MS/MS analysis (collision energy set at 20 eV). To analyse the mass spectra results, we used Agilent MassHunter software as reported previously [27].

### 2.7. Nucleotide Sequence Accession Number

The 16S rDNA sequence of strain T2-2 was assigned GenBank accession no. HQ907954. All other rDNA sequences were obtained from GenBank.

## 3. Results and Discussion

### 3.1. General Description and Molecular Identification of Strain T2-2

**Figure 1.**
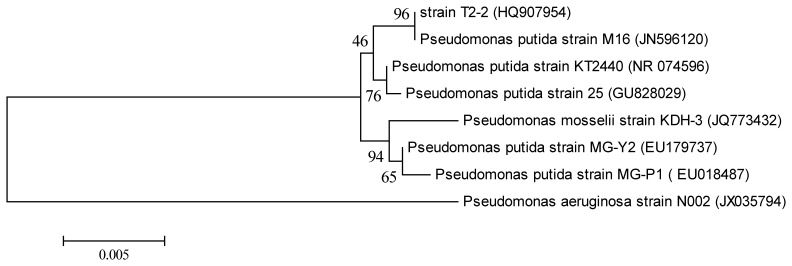
Phylogenetic analysis of oral bacterial strain T2-2. 16S rDNA-based phylogenetic tree showing the phylogenetic position of strain T2-2. A total of 1,529 unambiguously aligned nucleotides were analysed by using *MEGA* 5.2 as described in Materials and Methods. The barrepresents evolutionary distance as number of changes per nucleotide position, determined by measuring the lengths of the horizontal linesconnecting the corresponding species. *P. aeruginosa* strain N002 was used as outgroup. Numbers in parentheses are GenBank accession numbers.

The bacterial colony labelled as T2-2 was purified and its morphology was observed by growing on LB agar. Colonies of strain T2-2 appeared circular, with a size of 0.3 mm, with entire margin and opaque, raised colonies and milky white colour. Strain T2-2 was stained as Gram-negative and appeared as rod-shaped bacteria. Partial 16S rDNA sequences of strain T2-2 (1,529 nucleotides) were determined and phylogenetic analysis of strain T2-2 showed that it clustered closely (96%) to *P. putida* strain M16 (Figure 1). Hence, strain T2-2 was named as *P. putida* strain T2-2.

Evolutionary history was obtained by using Neighbour-Joining algorithm [53]. The percentage of the robustness of the associated taxa clustered together is indicated by the bootstrap test (1,000 replicates) and is shown beside the branch (Figure 1). The evolutionary distance can be determined as the tree is drawn to scale, and the evolutionary relationship of strains are indicated by the scale below. Evolutionary distances were computed using the Maximum Composite Likelihood method. Units used represent the number of base substitutions per site. Pairwise deletion is used in the construction in this tree and all positions containing alignment gaps and missing data were eliminated only in pairwise sequence comparison.

To our knowledge, there are no studies to date that have identified *P. putida* as part of the normal oral flora. *Pseudomonas* however can be normally found on the surfaces of plants and animals. The complete genome of the toluene-degrading *P. putida* strain KT2440 has been sequenced [54]. *P. putida* is non-pathogenic in nature due to the absence of virulence factors like certain exotoxin genes and type III secretion systems.

### 3.2. Detection of AHLs Production of T2-2 by Using Biosensor A. tumefaciens NTL4 (pZLR4)

**Figure 2.**
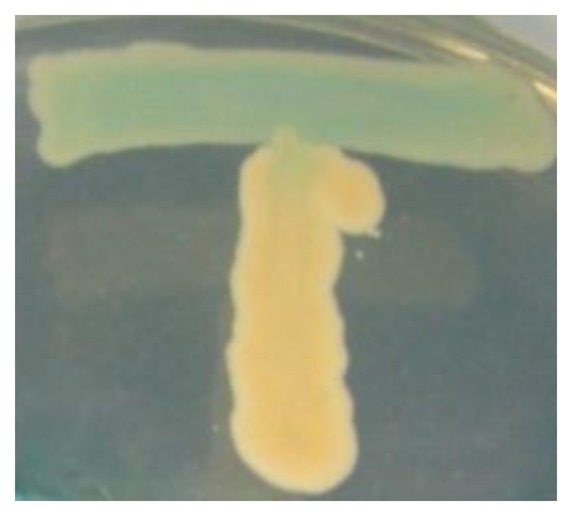
Cross streak of strain T2-2 colony with Agrobacterium tumefaciens NTL4 (pZLR4). Bacterial isolate T2-2 (vertical streak) were cross streaked with A. Tumefaciens NTL4 (pZLR4) (horizontal streak) to detect the presence of AHL production. Blue pigmentation indicated the presence of AHLs production by T2-2.

Bacterial isolates (vertical streak) were cross streaked with *A. tumefaciens* NTL4 (pZLR4) (horizontal streak) to detect the presence of AHL production. Strain T2-2 was able to induce synthesis of β-galactosidase by *A. tumefaciens*, which is indicated by the hydrolysis of X-gal depicted as blue pigmentation (Figure 2).

### 3.3. Measurement of Bioluminescence

**Figure 3.**
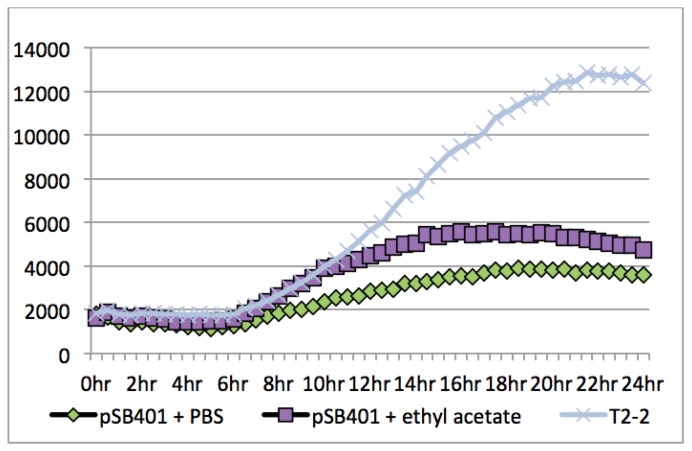
Measurement of bioluminescence. Bioluminescence and OD were measured throughout growth for 24 h at 37 °C in the presence of PBS buffer (diamond) and ethyl acetate (square) as negative controls, and AHL extract from strain T2-2 (*P. putida*) (asterisk). Each point was the average of data obtained from three experiments. Horizontal axis: time (hr, hour), vertical axis (RLU/OD_600 nm_ where RLU: relative light unit, OD: optical density at 600 nm).

To gain an insight of the type of AHLs produced, supernatant from strain T2-2 was extracted for AHLs assayed with biosensors used in conjunction with TLC. The TLC result revealed one well-resolved spot for strain T2-2 AHL extract. One spot had a relative migration factor (R*_f_*) similar to that of C6-HSL (data not shown). However, TLC is not a conclusive method to determine the exact identification of AHLs present in the extract. To further verify strain T2-2 did produce detectable AHLs, *lux*-based biosensor (*Escherichia coli* [pSB401]) was used to determine the AHLs inducible *lux* activity. Using *E. coli* [pSB401], it is shown that AHL extract of strain T2-2 induced bioluminescence over 24 h (Figure 3).

### 3.4. Mass Spectrometry Analysis of Oral Bacteria Spent Supernatants Extracts

**Figure 4.**
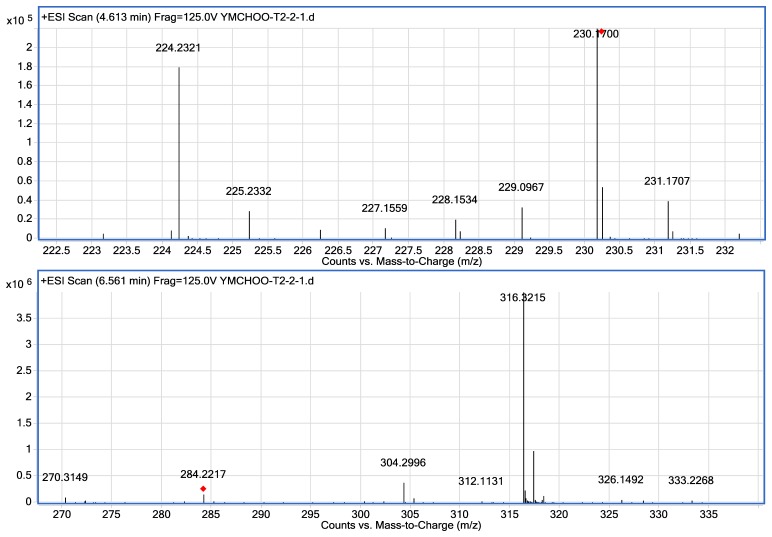
Mass spectrometry analysis of spent supernatants extract *P. putida* strain T2-2 **Upper panel**: mass spectra ofC8-HSL (*m/z* 228.1534) (marked by arrow), **lower panel**: C12-HSL (*m/z* 284.2217) (marked by arrow).

Mass spectrometry was used for unequivocal detection of AHLs produced by strain T2-2, the result of mass spectrometry confirmed the presence of C8-HSL (*m/z* 228.1534) and C12-HSL (284.2217) in the spent supernatant of strain T2-2. The ESI-MS/MS spectrum of C12-HSL shows fragments (*m/z* 95.0822, 109.1003) typical of an AHL lactone-moiety (Figure 4) [55].

Our group has previously reported the isolation of *K. pneumoniae* and *Enterobacter* sp. from oral cavity using KG medium [26,27]. Our previous work showed that these two oral bacteria exhibit QS properties and have shown to produce AHLs. We have also shown that *Enterobacter* sp. produces C8-HSL and C12-HSL [26]. Similarly, in this work, we showed that strain T2-2 also produced C8-HSL and C12-HSL (Figure 4). Surprisingly, the AHL production profile of *P. putida* strain ATCC 39168 is very different from our study. AHLs produced by *P. putida* strain ATCC 39168 included C6-HSL, C8-HSL, C10-HSL and 3-oxo-C12-HSL [55], whereby only C8-HSL is the common AHL produced by both *P. putida* strain T2-2 and *P. putida* strain 39168 [55]. This is possibly due to the different culturing conditions, where minimal medium was used in the previous study with glutamic acid (130 mg/L) was used as the sole carbon source [55], while LB medium was used in this study. It has been noted that using different culturing media would result in the production of differing AHLs [56]. In other reported work, other strains of *P. putida* have been shown to produce *N*-(3-oxo-dodecanoyl)-L-homoserine lactone (3-oxo-C12-HSL) [57,58]. However, strain T2-2 did not produce any detectable long chain 3-oxo-C12-HSL under our experimental conditions. Note that in the present work, bacterial strain T2-2 was grown aerobically in LB medium buffered with MOPS providing acidic growth condition because AHLs has a short half-life under alkaline conditions [48].

Reported work shows that *P. putida* strains WCS358 and IsoF [57,58] are beneficial rhizosphere bacteria that possess PpuI/R which are orthologues and responsible for the synthesis of and response to 3-oxo-C12-AHL. PpuI/R share approximately 50% with LasI/R of *P. aeruginosa*. Since *P. putida* is a plant-associated bacterium, we speculated that our strain T2-2 is originated from the vegetable diet consumed by the healthy individual before sampling. *P. putida* has been reported to produce AHL to regulate biofilm formation [57,58]. Hence, it is postulated that strain T2-2 may produce AHL to form biofilms on the tongue surface. Thus far, AHLs are detectable in body fluids and have been shown to exhibit immune modulatory effects *in vitro* [59], but the long term effects of ingesting AHLs on the digestive gastrointestinal tracts of human beings remain unknown.

This report represents the first documentation on the isolation of an AHL-producing *P. putida* strain T2-2 from the human tongue surface. Together with the previous finding on the presence of AHL- producing bacteria in the oral cavity, this work provides additional evidence to illustrate the importance to work on the AHL-producing bacteria present in the human oral cavity. Not only has this work expanded the scope of QS research in oral cavity, it also provides more evidence that QS bacteria relying on AHL as signaling molecules should not be underestimated.

## 4. Conclusions/Outlook

Here, oral *Pseudomonas* sp. strain T2-2 isolated from the human tongue surface has been shown to produce AHLs as confirmed by biosensors and high resolution tandem mass spectrometry. This work illustrates the importance of investigating the AHL-producing bacteria in the oral cavity.
